# Seizure, Deafness, and Renal Failure: A Case of Barakat Syndrome

**DOI:** 10.1155/2013/261907

**Published:** 2013-10-22

**Authors:** Nasrollah Maleki, Bahman Bashardoust, Manouchehr Iranparvar Alamdari, Zahra Tavosi

**Affiliations:** ^1^Department of Internal Medicine, Imam Khomeini Hospital, Ardabil University of Medical Sciences, Ardabil, Iran; ^2^Department of Internal Medicine, Shohadaye Khalije Fars Hospital, Bushehr University of Medical Sciences, Bushehr, Iran

## Abstract

Barakat syndrome (also known as HDR syndrome) is an autosomal dominant disorder characterized by hypoparathyroidism, sensorineural deafness, and renal disease caused by mutation of the GATA3 gene located at chromosome 10p15. The exact prevalence of this disorder is not known but is very rare, with only about a dozen cases reported in the literature. Here, we report a case of 58-year-old man from Ardabil who presented with seizure due to hypocalcemia. Further history revealed bilateral deafness. Audiogram confirmed sensorineural hearing loss of both sides. His laboratory data were consistent with hypoparathyroidism and renal failure. He was diagnosed to have Barakat syndrome based on his clinical and laboratory data. In conclusion, we need to be aware of rare inherited conditions in a patient with abnormal physical and laboratory findings even though their initial presentation was seizure and hypocalcemia.

## 1. Introduction

Barakat syndrome, also known as hypoparathyroidism, deafness, and renal dysplasia (HDR) syndrome, is a rare autosomal dominant disorder [1]. The syndrome was first noted in siblings with hypocalcemia and proteinuria [2]. Mutations in GATA3, a gene localized to the chromosome region 10p14-15, have been detected in families affected by the syndrome [3, 4]. GATA3 is a transcription factor that is involved in the embryonic development of the parathyroid glands, kidneys, inner ears, thymus, and central nervous findings. Similar clinical findings were reported in families with an apparent autosomal recessive mode of inheritance [5, 6].

In this report, we demonstrate a case of Barakat syndrome presented with seizure, hypoparathyroidism, and bilateral sensorineural deafness.

## 2. Case Report

**Figure 1:**
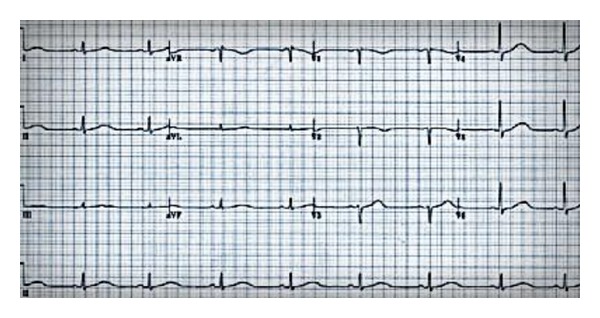
Electrocardiography showed a prolonged QT interval (560 msec).

A 58-year-old man from Ardabil was admitted to our hospital with generalized tonic-clonic seizure. The patient was a known case of chronic kidney disease 2 years ago. He had been experiencing recurrent episodes of seizure for 20 days before his admission. Further history revealed progressive hearing loss for the last 10 years but there was no developmental delay. He had a family history of kidney disease in siblings, which in their renal biopsy were reported to have FSGS. His vital signs were within the normal range. Neurological examination showed depression of deep tendon reflexes in both upper and lower extremities. Chvostek's and Trousseau's signs were negative. The initial laboratory studies showed serum calcium level: 5.3 mg/dL and serum phosphorus level: 7.2 mg/dL. The serum intact-PTH level measured by immunoradiometric assay (IRMA) was 5 ng/L (normal range: 15–60 ng/L). The patient did not have any history of neck surgery or radiation exposure that could have led to hypoparathyroidism. Other laboratory tests on the serum showed urea nitrogen: 30 mg/dL, creatinine: 2.5 mg/dL, albumin: 4.2 g/dL, sodium: 138 mEq/L, potassium: 3.9 mEq/L, and magnesium: 1.65 mg/dL (normal range: 1.6–2.3 mg/dL). Urine volume in 24 hours was 1700 mL with urine creatinine, protein, and calcium levels of 1020 mg/dL, 176 mg/dL, and 20 mg/dL. Erythrocyte sedimentation rate was 51 in the first hour and serum C-reactive protein level was 18 mg/dL. Serum protein electrophoresis was normal. Electrocardiography showed a prolonged QT interval (560 msec) (Figure 1).

**Figure 2:**
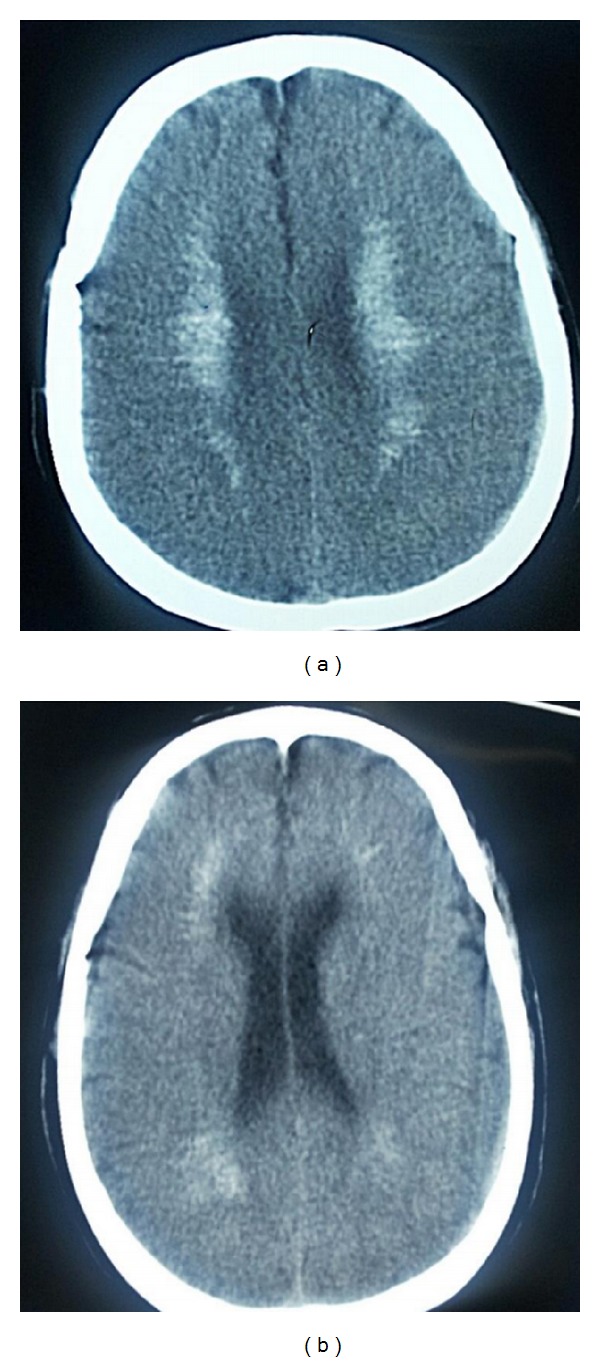
Noncontrast computed tomography of the brain showed intracranial calcification involving the basal ganglia, thalamus, and cerebral cortex on both the right (b) and left (a) sides.

**Figure 3:**
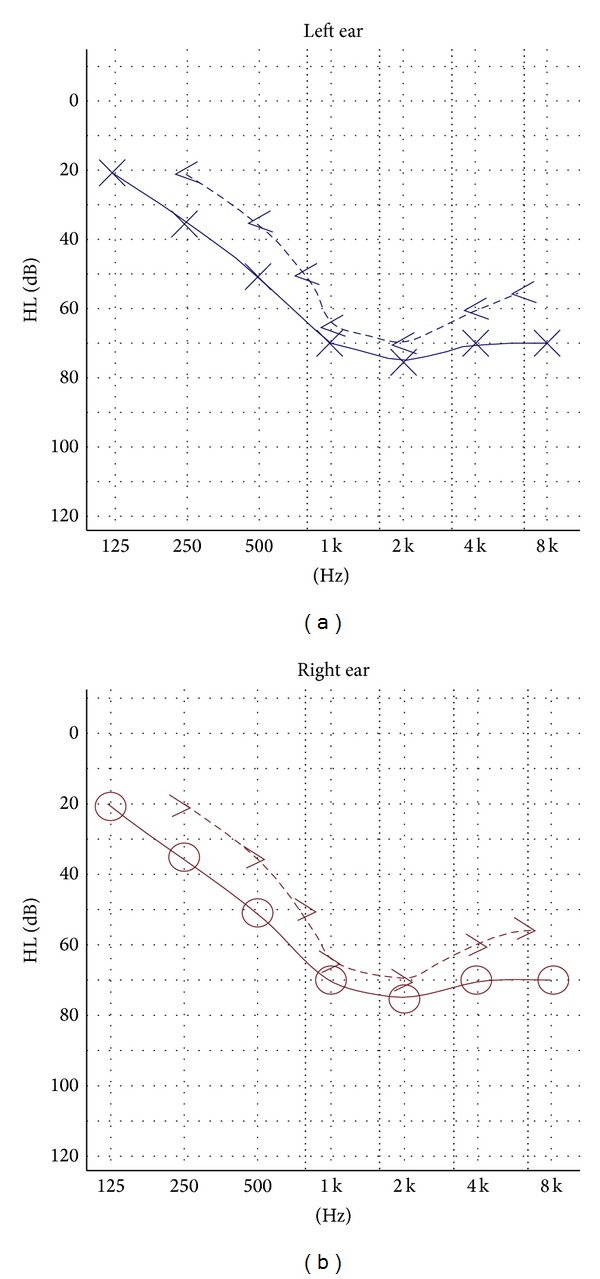
Pure tone audiometry showed bilateral moderate-to-severe hearing loss.

Noncontrast computed tomography of the brain showed intracranial calcification involving the basal ganglia, thalamus, and cerebral cortex on both the right and left sides (Figure 2). Pure tone audiometry showed bilateral moderate-to-severe hearing loss that was more severe at the higher end of the frequency spectrum (Figure 3). Abdominal ultrasonography revealed a reduction in size of both kidneys (80 mm in length) with an increased cortical echogenicity. Because of the simultaneous occurrence of hypoparathyroidism, deafness, and renal disease, the patient was diagnosed with HDR syndrome. However, chromosomal study of the patient using standard trypsin G-banding analysis showed no abnormality.

The patient was administered calcium carbonate, ergocalciferol, and rocaltrol. After the sizures were controlled, he was discharged and is being followed up.

## 3. Discussion

Barakat syndrome can occur at any age, and patients with this syndrome usually show symptoms related to hypocalcemia. It was first reported in 1977 by Barakat et al. [2]. In 1992, Bilous et al. [1] described two brothers and two daughters of one of the affected brothers with hypoparathyroidism, sensorineural deafness, and renal dysplasia. Subsequently, other reports appeared in the literature confirming that the syndrome is associated with a wide phenotype spectrum, consisting of hypoparathyroidism, sensorineural deafness, and renal disease [4, 7, 9, 13, 14]. Patients may present with hypocalcemia, tetany, or afebrile convulsions at any age. Hearing loss is usually bilateral (but may be asymmetric) and may range from mild to profound impairment, being more severe at the higher end of the frequency spectrum. Renal diseases in these patients include nephrotic syndrome, cystic kidney, renal dysplasia, hypoplasia or aplasia, pelvicalyceal deformity, vesicoureteral reflux, chronic renal failure, hematuria, proteinuria, and renal scarring [1, 2, 11, 15–17]. However, most patients show progression to chronic renal failure and require renal replacement therapy [4]. Our patient had chronic renal insufficiency. No renal dysplasia was detected on imaging studies. Deafness is a consistent feature of HDR syndrome. HDR patients have been reported to experience bilateral sensorineural deafness, which is more severe at higher frequencies [18]. Our patient also showed similar features on PTA.

The original patients described by Barakat et al. [2] presented with hypocalcemia and proteinuria, which progressed to a steroid resistant nephrotic syndrome, as well as with hypoparathyroidism and bilateral nerve deafness. Renal histology revealed fetal-like glomeruli and thickened glomerular basement membranes. The parathyroid glands were absent or fibrotic. All four siblings died with end-stage renal disease between the ages of 3 and 8. The patients reported by Bilous et al. [1] had bilateral renal dysplasia. Hasegawa et al. [7] found reports of 14 patients with deletion of 10p13; five had hypoparathyroidism or hypocalcemia, 6 had urinary tract abnormalities, and 2 had deafness. Fujimoto et al. [9] reported a Japanese boy with associated recurrent cerebral infarctions in the basal ganglia. Lichtner et al. [15] performed molecular deletion analysis of two patients with partial monosomy 10p and hypoparathyroidism, deafness and renal dysplasia, or renal insufficiency, cardiac defect, cleft palate, and reduced T-cell levels.  Table 1 showed characteristics of the reported cases of patients with HDR syndrome in the literature.

Diagnosis is based on the clinical finding. Diagnosis of suspected patients may be assisted by the following tests: measurement of PTH levels, an audiogram or auditory brain stem response study, renal imaging studies, and a renal biopsy. DNA analysis may demonstrate the presence of a submicroscopic deletion on chromosome 10p. Detailed study of chromosome 10p should be undertaken in patients with well-defined renal tract abnormality phenotypes, especially when there is associated hypoparathyroidism or deafness [3]. Various combinations of this syndrome have been reported including familial idiopathic hypoparathyroidism and progressive sensorineural deafness without renal disease [6, 8] and autosomal recessive hypoparathyroidism with renal insufficiency and developmental delay [5]. Treatment consists of treating the clinical abnormalities associated with hypoparathyroidism, deafness, and renal disease at the time of diagnosis. Prognosis depends on the nature and severity of the renal disease.

Based on the findings of the present case, it is highly recommended that, in the patients presenting with seizure associated with deafness, performing audiometry, calcium, phosphate, parathyroid hormone determination, and renal imaging, studies can probably reveal this extremely rare genetic disorder of Barakat syndrome.

## Figures and Tables

**Table 1:** Comparison of presentation for patients with HDR syndrome.

References	Symptoms	Heredity	Number of patients
Shaw et al. [5]	Hypoparathyroidism, renal failure, and developmental delay	Autosomal recessive	2 girls and 2 boys
Bilous et al. [1]	Deafness, hypoparathyroidism, and renal dysplasia	Autosomal dominant	2 sisters and 2 brothers
Hasegawa et al. [7]	Deafness, hypoparathyroidism, and renal dysplasia	Autosomal dominant	1 girl
Watanabe et al. [8]	Deafness, hypoparathyroidism, and without renal disease	Autosomal dominant	One-month-old infant and 5 members of family
Fujimoto et al. [9]	Deafness, hypoparathyroidism, renal dysplasia, and recurrent infarcts in basal ganglia	—	1 boy
Muroya et al. [4]	Deafness, hypoparathyroidism, and renal dysplasia	Autosomal dominant	9 patients
Aksoylar et al. [10]	Deafness, hypoparathyroidism, renal dysplasia, and psoriasis	Autosomal dominant	An 18-year-old girl
Kato et al. [11]	Deafness, hypoparathyroidism, renal dysplasia, nephrocalcinosis, and renal tubular acidosis	Autosomal dominant	A 34-year-old woman
Taslipinar et al. [12]	Deafness, hypoparathyroidism, renal dysplasia, and renal tubular acidosis	Autosomal dominant	A 19-year-old man
